# Study on the relationship between body mass index and blood pressure indices in children aged 7–17 during COVID-19

**DOI:** 10.3389/fpubh.2024.1409214

**Published:** 2024-06-19

**Authors:** SuJie Mao, GuoPing Qian, KaiWen Xiao, Hong Xu, Hao Zhou, XiuJin Guo

**Affiliations:** ^1^Graduate Development, Harbin Sport University, Harbin, Heilongjiang, China; ^2^Faculty of Sports Medicine, Gdansk University of Sport, Gdańsk, Poland; ^3^Discipline Development Office, Nanjing Sport Institute, Nanjing, Jiangsu, China; ^4^College of Sports and Health, Sangmyung University, Seoul, Republic of Korea; ^5^Teaching Evaluation Center, Nanjing Police University, Nanjing, Jiangsu, China

**Keywords:** children, physical fitness, BMI, predictive model, obesity

## Abstract

**Background:**

To explore the relationship between body mass index (BMI), age, sex, and blood pressure (systolic blood pressure, SBP; diastolic blood pressure, DBP) in children during COVID-19, providing reference for the prevention and screening of hypertension in children.

**Methods:**

This study adopted a large-scale cross-sectional design to investigate the association between BMI and blood pressure in 7-17-year-old students in City N, China, during COVID-19. Thirty-six primary and secondary schools in City N were sampled using a stratified cluster sampling method. A total of 11,433 students aged 7–17 years in City N, China, were selected for blood pressure (Diastolic blood pressure, DBP, Systolic blood pressure, SBP), height, and weight, Resting heart rate (RHR), chest circumference, measurements, and the study was written using the STROBE checklist. Data analysis was conducted using SPSS 26.0, calculating the mean and standard deviation of BMI and blood pressure for male and female students in different age groups. Regression analysis was employed to explore the impact of BMI, age, and sex on SBP and DBP, and predictive models were established. The model fit was evaluated using the model R^2^.

**Results:**

The study included 11,287 primary and secondary school students, comprising 5,649 boys and 5,638 girls. It was found that with increasing age, BMI and blood pressure of boys and girls generally increased. There were significant differences in blood pressure levels between boys and girls in different age groups. In regression models, LC, Age, BMI, and chest circumference show significant positive linear relationships with SBP and DBP in adolescents, while RHR exhibits a negative linear relationship with SBP. These factors were individually incorporated into a stratified regression model, significantly enhancing the model’s explanatory power. After including factors such as Age, Gender, and BMI, the adjusted R^2^ value showed a significant improvement, with Age and BMI identified as key predictive factors for SBP and DBP. The robustness and predictive accuracy of the model were further examined through K-fold cross-validation and independent sample validation methods. The validation results indicate that the model has a high accuracy and explanatory power in predicting blood pressure in children of different weight levels, especially among obese children, where the prediction accuracy is highest.

**Conclusion:**

During COVID-19, age, sex, and BMI significantly influence blood pressure in children aged 7–17 years, and predictive models for SBP and DBP were established. This model helps predict blood pressure in children and reduce the risk of cardiovascular diseases. Confirmation of factors such as sex, age, and BMI provide a basis for personalized health plans for children, especially during large-scale infectious diseases, providing guidance for addressing health challenges and promoting the health and well-being of children.

## 1 Introduction

Hypertension is one of the largest disease burdens globally (1). According to WHO statistics, in 2010, approximately 1.4 billion people worldwide suffered from hypertension, and it is projected to reach 1.6 billion by 2025 (2). In China, the number of hypertensive patients is close to 330 million, with about one in every four individuals affected (3). Moreover, with the improvement of living standards, there is a trend toward “youthfulness” in the prevalence of hypertension, with more and more children affected. According to the Chinese Center for Disease Control and Prevention, the proportion of high blood pressure among Chinese children is on the rise, reaching approximately 15%, and this trend continues to increase. Hypertension in children not only poses problems for their development but also causes damage to various organs in their bodies (4). Previous studies have shown that children with hypertension are 4.6 times more likely to develop hypertension in adulthood than non-hypertensive children (5, 6). Hypertension in children is mainly divided into primary and secondary hypertension (7). In the adolescent population, primary hypertension is mainly associated with factors such as genetics, diet, and obesity. Numerous studies have shown that obese adolescent children have a 3.5 times higher risk of developing hypertension than non-obese children (8–10).

Hypertension in children imposes a significant burden on the heart and vascular system, leading to excessive pressure on the heart, thereby affecting its normal development and function (11). Long-term hypertension may result in cardiac hypertrophy and myocardial dysfunction, affecting the heart’s ability to pump blood effectively (12). The increase in blood pressure caused by childhood hypertension also increases the risk of damage and hardening of blood vessel walls, reducing vascular elasticity and function (13), thereby reducing the ability to regulate blood flow and exacerbating hypertension. Hypertension may also impair renal function in children, increasing the risk of chronic kidney disease and affecting metabolic and excretory functions, thereby affecting overall health development (14, 15). Additionally, hypertension in children may lead to a series of complications, affecting the normal development of the brain cortex and neurons, thereby affecting intellectual development and cognitive abilities (16). Long-term discomfort (such as headaches, dizziness, etc.) may lead to emotional problems, including anxiety, depression, and other mental health issues, thereby affecting their normal social and interpersonal relationship development (17). Therefore, blood pressure prevention and control during adolescence are particularly important.

During the COVID-19 pandemic, the proportion of hypertension in children is increasing (18–20). This phenomenon may be related to lifestyle and behavioral changes brought about by the epidemic, including social restrictions, distance learning, and reduced indoor activities (21). Due to the influence of epidemic control measures such as lockdowns, children may be more prone to unhealthy habits, such as prolonged use of electronic devices, lack of physical exercise, and irregular diet (22). These factors not only increase the obesity rate in children but also increase the risk of hypertension in children.

Body Mass Index (BMI) is a measure of body mass relative to height, commonly used to estimate body fatness, calculated as weight/height^2^ (kg/cm^2^), and widely used in testing, research, and surveys (23). It is not only used to assess obesity but can also serve as an indicator for predicting cardiovascular diseases (24). Numerous studies have shown that as BMI levels increase, the incidence of hypertension tends to rise (25). Controlling BMI levels can effectively reduce the incidence of hypertension and protect cardiovascular health (26). A recent study found that during the COVID-19 quarantine period, both boys and girls showed an increasing trend in blood pressure with increasing BMI, and obesity is a potential risk factor for hypertension (27). A large body of research has confirmed that the blood pressure of adolescents is closely related to a variety of physiological indicators (28–30). Although these findings provide important insights into understanding the changes in blood pressure among adolescents, there is still a lack of research in this field that constructs and validates specific predictive models using large-sample data, which limits the generalizability and application of related models. This study constructs a blood pressure prediction model that includes physiological parameters, Age, and BMI, and performs cross-validation to verify the correlation between blood pressure and factors such as BMI and Age. It explores the changing patterns of these correlations in different Gender, obesity levels, and Age stages, as well as how these variables collectively influence blood pressure levels. We aim to establish a reliable computational model for the relationship between BMI and blood pressure. By identifying high-risk groups early, targeted health education and intervention measures can be implemented to guide children to adopt healthy lifestyles, including balanced diets, moderate exercise, reducing the link between obesity and hypertension, and reducing the risk of hypertension and related complications in children. At the same time, it also provides a scientific basis for the formulation of public health policies, promoting comprehensive attention to and protection of the health of children.

## 2 Materials and methods

This study employed a large-scale cross-sectional design, targeting students from the second grade of primary school to the second grade of high school (ages 7–17) in City N. The STROBE checklist was utilized to ensure reporting transparency (31). The aim was to investigate the association between BMI and blood pressure among children during the COVID-19 period and establish models relating gender, age, height, weight, and blood pressure.

### 2.1 Study subjects

The investigation targeted Han Chinese students aged 7 to 17 in urban and rural areas of Nanjing City in 2022, ranging from the second grade of primary school to the second grade of high school. A stratified cluster sampling method was employed during the monitoring process. Stratification was based on grade level, and random cluster sampling was conducted at the class level to ensure the representativeness of the sample. Thirty-six schools were selected from all primary and secondary schools in City N, covering 12 districts. Each district randomly chose one primary school (ages 7–11), one junior high school (ages 12–14), and one high school (ages 15–17, including directly affiliated schools). A total of 11,427 individuals were tested. The sample was grouped by age into 11 age brackets ranging from 7 to 17 years old, with each age bracket including both boys and girls, resulting in a total of 22 age groups.

### 2.2 Inclusion and exclusion criteria

Inclusion criteria:

1. Han Chinese urban and rural male and female students aged between 7 and 17 years old.
2. Students enrolled in grades two to high school second year in City N, China in 2021.
3. Students whose families agreed to participate in the study and signed informed consent forms.
4. Students in good health without serious illnesses or disabilities.

Exclusion criteria:

1. Physical measurement samples were selected from students participating in health examinations, comprising normal students (defined as students capable of participating in various physical exercise activities, with sound development and good health).
2. Individuals with major organ diseases affecting the heart, liver, spleen, or kidneys, physical deformities, or acute illnesses were excluded.
3. Female students during menstruation were excluded from the testing project.
4. Classes for students with sports specialties were excluded.

### 2.3 Data testing

Data collection for this study strictly followed the “Chinese National Physical Fitness Monitoring Manual” to ensure the scientific and standardized monitoring. All personnel participating in testing and assessment received systematic training on the Chinese national student physical fitness health standards to ensure they possessed sufficient professional knowledge and skills. The training content strictly followed the testing data requirements and procedures of the Physical Education, Health and Art Department of the Chinese Ministry of Education, ensuring the scientific, standardized, and accurate testing. All testing content and research procedures strictly adhered to the ethical principles and research norms stipulated in the Helsinki Declaration (32) and were approved by the Ethics Committee of the Sports Human Body Science Research Institute of Nanjing Sport Institute, with operations conducted strictly in accordance with its guidance and review opinions.

### 2.4 Data collection

Each student participating in the test was given a physical monitoring card, which was used to record individual age, gender, height, weight, systolic blood pressure (SBP), and diastolic blood pressure (DBP). After the test was completed, the monitoring card was collected, and data entry was performed using EXCEL. This standardized data collection process helps ensure the accuracy and consistency of the data and provides a reliable foundation for subsequent data analysis.

### 2.5 Data cleaning and screening

Systematic processing of data was conducted using EXCEL software in this study. Potential outliers and incorrectly entered data were identified to ensure the reliability of subsequent statistical analysis. During this process, special attention was paid to identifying outliers and checking erroneous data, deleting or correcting non-standard data points that may affect the study conclusions.

### 2.6 Data analysis

After conducting a secondary screening of the recorded data, we employed the statistical tool SPSS 26.0 to perform descriptive statistical analysis on student height, weight, age, gender, SBP, DBP, RHR, chest circumference, and LC. We calculated the mean and standard deviation of BMI and blood pressure for males and females in each age group to assess their average levels and distributions. Following the Chinese Physical Health Standards for Primary and Secondary School Students (33), we assigned weighted values to participants based on their age, gender, and BMI, and categorized them into four groups: underweight, normal weight, obese, and overweight according to their BMI scores. Based on these BMI categories, we calculated predictive models for blood pressure in adolescents and children. To ensure the accuracy and generalizability of the regression models, we randomly selected a sample size equivalent to 20 times the inclusion criteria for model validation, thereby assessing the practical application of the models in adolescents and children.

Through correlation analysis, we evaluated the linear relationship between age, gender, BMI, chest circumference, RHR, LC, and other indicators with blood pressure in adolescents and children. Stratified regression analysis was conducted based on the variables exhibiting a linear relationship. By observing the variance inflation factor (VIF) in the stratified collinearity diagnosis, we determined the indicators that should be included in the regression models. Regression models were constructed separately for different groups based on BMI categories, allowing for targeted predictions of blood pressure levels in different physical contexts. Using R 4.3.1, we performed K-fold cross-validation and tested the models using additional independent samples. The accuracy, stability, and reliability of the models were further validated by calculating the mean squared error (MSE), root mean squared error (RMSE), and mean absolute error (MAE) between the predicted and measured blood pressure values.

## 3 Results

### 3.1 Descriptive statistics and weighted analysis

#### 3.1.1 Overview of data collection

In total, data from 14,333 primary and secondary school students were collected in this study. After careful screening and processing of the data, records containing outliers and missing values were excluded, reducing the final effective dataset to 11,407 students. To ensure the stability and reliability of the established model, a further random sample of 120 students was selected from the effective dataset as a validation sample for the model. The total number of students actually participating in the data analysis was finally determined to be 11,287, including 5,649 boys and 5,638 girls, with ages ranging from 7 to 17 years old. These samples will be used in subsequent analyses to evaluate and establish predictive models for blood pressure in children and adolescents.

#### 3.1.2 Weighting and classification

Based on the Chinese Physical Health Standard for Primary and Middle School Students, we scored and rated the Body Mass Index (BMI) of the participants. Using BMI scoring tables specific to different ages and genders, each student’s BMI value was assigned a corresponding score ranging from 60 to 100. These scores then categorized students into four weight classes: normal weight, underweight, overweight, and obese. This classification allowed for a more precise consideration of the potential impact of weight factors on blood pressure, enhancing the accuracy of the model in the prediction process.

#### 3.1.3 Sample characteristics description

In this study, BMI and blood pressure data from 11,287 valid samples were analyzed. Among these, 7,898 students were of normal weight, 165 were underweight, 1,859 were overweight, and 1,365 were obese. Detailed data classified by age and gender revealed the distribution patterns of BMI across different categories, assessing students’ health status and preventing potential health issues. Simultaneously, blood pressure measurements uncovered trends with age, possibly reflecting the natural physiological development of adolescents.

**Figure 1:**
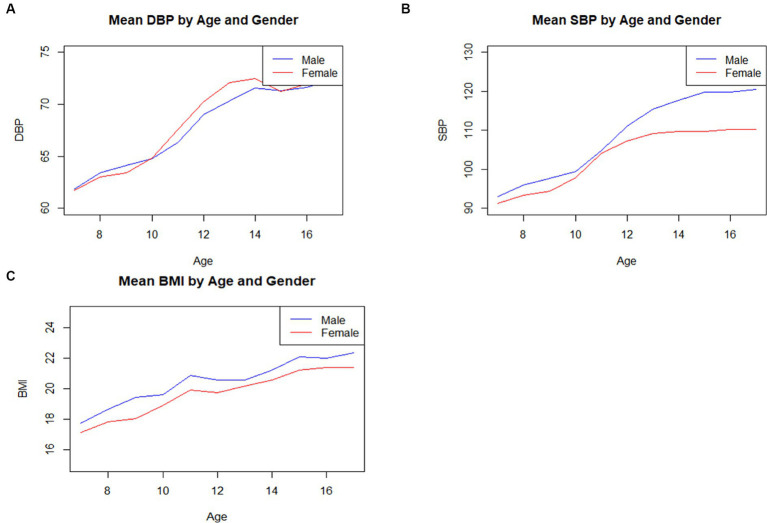
The fluctuating patterns of SBP, DBP, BMI indicators among boys and girls aged 7–17. **(A)** The fluctuating patterns of SBP, DBP, BMI indicators among boys and girls aged 7–17. **(B)** The evolving patterns of SBP indicators among boys and girls aged 7–17. **(C)** The shifting patterns of BMI indicators among boys and girls aged 7–17.

According to Table 1 and Figure 1, it can be observed that the average BMI for both genders tends to increase with age. For instance, the average BMI for 7-year-old boys is 17.75, which rises to 22.40 by age 17. Similarly, girls’ BMI increases from 17.16 at age 7 to 21.41 at age 17. Additionally, blood pressure indicators, including SBP and DBP, show a similar upward trend with age.

**Table 1:** Basic characteristics description of adolescents and children of different ages and genders.

Age (year)	Gender	Value	BMI (kg/m^2^)	CC (cm)	RHR (bpm)	LC (L)	SBP (mmHg)	DBP (mmHg)
7	1	Mean	17.75	63.14	89.61	1372.17	93.07	61.83
		SD	2.64	5.52	9.15	402.13	9.29	7.58
	2	Mean	17.16	61.61	90.89	1288.16	91.35	61.75
		SD	2.42	5.44	9.98	259.45	10.24	8.04
8	1	Mean	18.69	66.32	88.68	1573.74	95.96	63.43
		SD	3.12	6.51	9.57	329.84	9.77	7.53
	2	Mean	17.84	64.47	89.86	1482.58	93.32	63.03
		SD	3.04	6.13	9.33	289.27	9.60	7.67
9	1	Mean	19.45	69.09	87.31	1792.09	97.66	64.11
		SD	3.53	7.41	9.45	401.10	9.51	7.49
	2	Mean	18.06	67.36	89.26	1669.85	94.38	63.41
		SD	2.89	6.65	9.43	356.90	9.70	7.23
10	1	Mean	19.62	70.92	85.04	2086.68	99.52	64.77
		SD	3.32	7.33	9.93	486.41	10.59	7.55
	2	Mean	18.92	71.90	87.07	2031.50	97.85	64.81
		SD	3.03	7.71	9.83	523.67	10.19	7.18
11	1	Mean	20.87	74.80	87.40	2377.74	104.83	66.38
		SD	3.79	8.51	9.58	500.39	11.20	7.66
	2	Mean	19.96	76.69	89.59	2233.56	104.10	67.58
		SD	3.32	7.90	9.93	434.85	10.98	7.29
12	1	Mean	20.63	75.07	83.35	3112.15	111.16	69.04
		SD	3.78	8.79	8.52	658.49	11.21	7.94
	2	Mean	19.76	78.75	83.13	2684.51	107.31	70.28
		SD	3.02	7.06	8.67	465.13	10.16	7.52
13	1	Mean	20.56	77.42	79.49	3597.98	115.52	70.36
		SD	3.62	7.30	9.60	683.60	9.99	8.22
	2	Mean	20.20	80.36	81.98	2827.50	109.26	72.10
		SD	2.86	6.68	8.82	452.83	10.10	8.21
14	1	Mean	21.21	80.84	80.36	3809.15	117.64	71.55
		SD	3.85	8.46	9.77	753.38	8.88	7.91
	2	Mean	20.63	81.59	82.95	3016.68	109.64	72.49
		SD	3.22	7.72	9.20	610.03	10.37	7.82
15	1	Mean	22.11	81.53	80.17	4141.50	119.99	71.31
		SD	3.55	7.86	9.37	692.64	7.87	8.44
	2	Mean	21.27	83.61	82.34	2967.70	109.73	71.24
		SD	2.97	6.47	8.67	505.94	10.50	7.13
16	1	Mean	22.00	82.78	79.47	4312.29	119.79	71.60
		SD	3.16	6.96	9.10	722.14	7.55	7.48
	2	Mean	21.45	84.23	82.38	3067.28	110.34	71.97
		SD	3.03	6.52	8.58	508.45	10.31	7.21
17	1	Mean	22.40	84.63	80.26	4440.10	120.66	72.33
		SD	3.16	7.10	9.88	722.74	7.92	7.44
	2	Mean	21.41	84.14	83.83	3139.38	110.18	72.31
		SD	2.79	6.05	8.50	547.68	10.31	7.55

CC, Chest circumference; RHR, Resting Heart Rate; LC, Lung capacity; SBP, Systolic blood pressure; DBP, Diastolic blood pressure.

### 3.2 Correlation analysis results

As shown in Table 2, there is a strong positive linear correlation between LC and both SBP (correlation coefficient of 0.646) and DBP (correlation coefficient of 0.365). Age also exhibits relatively high correlation coefficients with SBP (0.619) and DBP (0.429), confirming the upward trend of blood pressure with age. In contrast, Gender has a minor influence on blood pressure, showing a slight negative linear relationship with SBP (−0.193). BMI and chest circumference also demonstrate significant positive linear relationships with blood pressure. The correlation coefficients between BMI and SBP, DBP are 0.472 and 0.290, respectively, while those for chest circumference are 0.595 and 0.421. This suggests that blood pressure increases with rising weight and chest circumference. RHR has a negative linear relationship with SBP (−0.162) but a weaker influence on DBP (0.052), indicating that an increase in RHR may be somewhat associated with adolescent blood pressure.

**Table 2:** Correlation coefficients of various variables with blood pressure in adolescents and children.

SBP Pearson correlation coefficient		DBP Pearson correlation coefficient
Age	0.619**	0.429**
Gender	−0.193**	0.025**
BMI	0.472**	0.290**
Chest circumference	0.595**	0.421**
Resting Heart Rate	−0.162**	0.052**
Lung capacity	0.646**	0.365**

*p < 0.05; **p < 0.01.

### 3.3 Hierarchical regression analysis

#### 3.3.1 Model construction

To delve deeper into the relationship between BMI and SBP among adolescents, a hierarchical regression model was employed. The data was stratified into six levels, and variables such as Age, Gender, chest circumference, RHR, and LC were progressively introduced into the model. This approach allowed for a layered analysis of the combined effects of these factors on SBP and DBP in adolescents and children, enhancing the model’s explanatory power. To ensure model accuracy and avoid multicollinearity issues, a collinearity diagnosis was performed by calculating the variance inflation factor (VIF) for each variable (a VIF value exceeding 5 suggests potential collinearity problems). This ensured the model’s precision and interpretability while accurately predicting trends in adolescent SBP.

#### 3.3.2 Hierarchical regression model results

As shown in Table 3, the initial model incorporating Age demonstrated a significant impact on SBP (adjusted R-squared of 0.385). The model’s explanatory power increased as Gender, BMI, chest circumference, RHR, and LC were gradually introduced, raising the adjusted R-squared for SBP to 0.504. The analysis for DBP began with a basic model including Age (adjusted R-squared of 0.185), and subsequent variable additions improved the model’s explanatory power to 0.254. All variables were significant at each model level (*p* < 0.01). Age and BMI emerged as key predictors for both SBP and DBP, with LC significantly influencing the final layer of the SBP model.

**Table 3:** Regression model results for blood pressure analysis.

SBP Stratified Regression analysis results							DBP Stratified Regression analysis results					
	Stratified1	Stratified2	Stratified3	Stratified4	Stratified5	Stratified6	Stratified1	Stratified2	Stratified3	Stratified4	Stratified5	Stratified6
Constant	74.078** (188.173)	81.983** (171.103)	66.668** (102.927)	61.449** (82.475)	54.120** (46.304)	55.503** (48.201)	54.017** (188.217)	53.367** (148.033)	47.554** (94.163)	43.971** (75.549)	27.307** (30.587)	27.462** (30.712)
Age	2.666** (83.903)	2.668** (86.679)	2.248** (70.413)	1.823** (41.315)	1.903** (42.208)	1.264** (22.954)	1.172** (50.591)	1.172** (50.602)	1.013** (40.672)	0.721** (20.909)	0.903** (26.205)	0.831** (19.440)
Gender		−5.282** (−27.244)	−4.529** (−24.306)	−5.171** (−27.141)	−5.338** (−27.935)	−3.560** (−17.066)		0.434** (2.978)	0.720** (4.959)	0.280 (1.881)	−0.100 (−0.685)	0.099 (0.613)
BMI			0.957** (33.369)	0.416** (8.592)	0.405** (8.393)	0.422** (8.890)			0.363** (16.245)	−0.008 (−0.219)	−0.032 (−0.881)	−0.031 (−0.830)
CC				0.293** (13.825)	0.296** (13.972)	0.210** (9.872)				0.201** (12.148)	0.207** (12.780)	0.197** (11.929)
RHR					0.079** (8.122)	0.096** (10.052)					0.179** (24.177)	0.181** (24.343)
LC						0.003** (19.620)						0.000** (2.831)
Sample size	11247	11247	11247	11247	11247	11247	11247	11247	11247	11247	11247	11247
*R* ^2^	0.385	0.423	0.475	0.484	0.487	0.504	0.185	0.186	0.205	0.215	0.254	0.254
Adjusted*R*^2^	0.385	0.423	0.475	0.484	0.487	0.504	0.185	0.186	0.205	0.215	0.253	0.254
*F*	*F* (1,11245) = 7039.676, *p* = 0.000	*F* (2,11244) = 4122.966, *p* = 0.000	*F* (3,11243) = 3391.773, *p* = 0.000	*F* (4,11242) = 2634.629, *p* = 0.000	*F* (5,11241) = 2133.076, *p* = 0.000	*F* (6,11240) = 1902.434, *p* = 0.000	*F* (1,11245) = 2559.408, *p* = 0.000	*F* (2,11244) = 1285.034, *p* = 0.000	*F* (3,11243) = 964.686, *p* = 0.000	*F* (4,11242) = 769.840, *p* = 0.000	*F* (5,11241) = 764.748, *p* = 0.000	*F* (6,11240) = 639.024, *p* = 0.000
△*R*^2^	0.385	0.038	0.052	0.009	0.003	0.017	0.185	0.001	0.019	0.010	0.039	0.001
△*F*	*F* (1,11245) = 7039.676, *p* = 0.000	*F* (1,11244) = 742.227, *p* = 0.000	*F* (1,11243) = 1113.513, *p* = 0.000	*F* (1,11242) = 191.127, *p* = 0.000	*F* (1,11241) = 65.965, *p* = 0.000	*F* (1,11240) = 384.940, *p* = 0.000	*F* (1,11245) = 2559.408, *p* = 0.000	*F* (1,11244) = 8.868, *p* = 0.003	*F* (1,11243) = 263.901, *p* = 0.000	*F* (1,11242) = 147.573, *p* = 0.000	*F* (1,11241) = 584.540, *p* = 0.000	*F* (1,11240) = 8.016, *p* = 0.005

Dependent variable: SBP, DBP. *p < 0.05, **p < 0.01. The value in parentheses is the *t*-value. CC, Chest circumference; RHR, Resting Heart Rate; LC, Lung capacity.

A collinearity diagnosis was conducted on the blood pressure prediction variables for adolescents using a multiple regression model (Table 4). The results indicated that the VIF values for Age, BMI, Gender, RHR, and LC did not exceed the threshold of 5, justifying their inclusion in blood pressure prediction models for different BMI categories. However, the high VIF value for chest circumference in both SBP and DBP models suggested strong collinearity with other variables. Therefore, to ensure model stability and interpretability, chest circumference was excluded from the regression model.

**Table 4:** Presents the collinearity diagnosis of predictor variables for adolescent children.

SBP Collinearity diagnostic table (VIF)							DBP Collinearity diagnostic table (VIF)					
Term	Stratified 1	Stratified 2	Stratified 3	Stratified 4	Stratified 5	Stratified 6	Stratified 1	Stratified 2	Stratified 3	Stratified 4	Stratified 5	Stratified 6
Age	1.000	1.000	1.183	2.298	2.413	3.719	1.000	1.000	1.183	2.298	2.413	3.719
Gender	–	1.000	1.015	1.079	1.092	1.345	–	1.000	1.015	1.079	1.092	1.345
BMI	–	–	1.198	3.468	3.470	3.471	–	–	1.198	3.468	3.470	3.471
CC	–	–	–	5.609	5.610	5.857	–	–	–	5.609	5.610	5.857
RHR	–	–	–	–	1.128	1.138	–	–	–	–	1.128	1.138
LC	–	–	–	–	–	3.628	–	–	–	–	–	3.628

CC, Chest circumference; RHR, Resting Heart Rate; LC, Lung capacity.

### 3.4 Model prediction and validation

#### 3.4.1 Model prediction

In this study, prediction models for SBP and DBP were designed for children of different weight categories. For normal-weight children (Table 5), the SBP model considered factors such as Age, Gender, BMI, RHR, and LC, explaining 49.5% of the variation and performing significantly in an F-test (*F* = 1541.895, *p* < 0.001). The DBP model for this category was also significant, explaining 25.5% of the variation (*F* = 538.487, *p* < 0.001). For underweight children (Table 6), the SBP model had an explanatory power of 32.4% (*R*^2^ = 0.324) and was significant in the F-test (*F* = 15.118, *p* < 0.001). The DBP model explained 23.1% of the variation (*R*^2^ = 0.231) and passed the F-test (*F* = 9.479, *p* < 0.001). Both models had VIF values less than 5, indicating no collinearity issues, and a D-W value of approximately 2, suggesting no autocorrelation in the models. For overweight children (Table 7), the SBP model explained 51.0% of the variation (*F* = 383.782, *p* < 0.001), while the DBP model explained 21.9% of the variation (*F* = 103.438, *p* < 0.001). The obesity models explained 43.9 and 20.3% of the variation in SBP and DBP predictions, respectively (Table 8), both passing the F-test (SBP: *F* = 212.273, *p* < 0.001; DBP: *F* = 69.044, *p* < 0.001). Although the VIF value for the overweight children’s model was slightly higher, suggesting possible mild collinearity, all models had D-W values close to 2, further confirming the absence of autocorrelation.

**Table 5:** Linear regression analysis of normal weight blood pressure.

Normal weight SBP linear regression analysis results (*n* = 7,882)								Normal weight DBP linear regression analysis results (*n* = 7,882)						
	Non-standardized coefficient		Standardized coefficient	*t*	*p*	Collinearity diagnosis		Non-standardized coefficient		Standardized coefficient	*t*	*p*	Collinearity diagnosis	
	*B*	Standard Error	*Beta*			VIF	Tolerance	*B*	Standard Error	*Beta*			VIF	Tolerance
Constant	53.064	1.524	–	34.814	0.000**	–	–	31.473	1.178	–	26.714	0.000**	–	–
Age	1.429	0.065	0.332	21.934	0.000**	3.563	0.281	1.092	0.050	0.398	21.690	0.000**	3.563	0.281
Gender	−3.101	0.241	−0.114	−12.881	0.000**	1.230	0.813	0.678	0.186	0.039	3.646	0.000**	1.230	0.813
BMI	1.106	0.070	0.175	15.889	0.000**	1.891	0.529	0.257	0.054	0.064	4.780	0.000**	1.891	0.529
RHR	0.108	0.012	0.079	9.280	0.000**	1.142	0.876	0.175	0.009	0.203	19.503	0.000**	1.142	0.876
LC	0.004	0.000	0.277	18.811	0.000**	3.377	0.296	0.001	0.000	0.107	5.974	0.000**	3.377	0.296
*R* ^2^	0.495							0.255						
Adjusted *R*^2^	0.494							0.254						
*F*	*F* (5,7876) = 1541.895, *p* = 0.000							*F* (5,7876) = 538.487, *p* = 0.000						
D-W	1.988							1.964						

Dependent variable: SBP, DBP; *p < 0.05, **p < 0.01, CC, Chest circumference; RHR, Resting Heart Rate; LC, Lung capacity.

**Table 6:** Linear regression analysis of low weight blood pressure.

Linear SBP linear regression analysis results (*n* = 164)								Linear DBP linear regression analysis results (*n* = 164)						
	Non-standardized coefficient		Standardized coefficient	*t*	*p*	Collinearity diagnosis		Non-standardized coefficient		Standardized coefficient	*t*	*p*	Collinearity diagnosis	
	*B*	Standard Error	*Beta*			VIF	Tolerance	*B*	Standard Error	*Beta*			VIF	Tolerance
Constant	85.761	12.349	–	6.945	0.000**	–	–	38.947	10.308	–	3.779	0.000**	–	–
Age	2.457	0.601	0.534	4.089	0.000**	3.982	0.251	1.744	0.501	0.484	3.477	0.001**	3.982	0.251
Gender	−3.379	1.936	−0.135	−1.745	0.083	1.388	0.720	−0.267	1.616	−0.014	−0.165	0.869	1.388	0.720
BMI	−1.195	0.826	−0.133	−1.447	0.150	1.961	0.510	−0.621	0.689	−0.088	−0.901	0.369	1.961	0.510
RHR	0.054	0.091	0.039	0.592	0.555	1.037	0.964	0.173	0.076	0.161	2.268	0.025*	1.037	0.964
LC	0.002	0.002	0.103	0.805	0.422	3.792	0.264	0.001	0.002	0.064	0.474	0.636	3.792	0.264
*R* ^2^	0.324							0.231						
Adjusted *R*^2^	0.302							0.206						
*F*	*F* (5,158) = 15.118, *p* = 0.000							*F* (5,158) = 9.479, *p* = 0.000						
D-W	2.048							1.906						

Dependent variable: SBP, DBP. *p < 0.05, **p < 0.01, CC, Chest circumference; RHR, Resting Heart Rate; LC, Lung capacity.

**Table 7:** Linear regression analysis of overweight blood pressure.

Overweight SBP linear regression analysis results (*n* = 1,848)								Overweight DBP linear regression analysis results (*n* = 1,848)						
	Non-standardized coefficient		Standardized coefficient	*t*	*p*	Collinearity diagnosis		Non-standardized coefficient		Standardized coefficient	*t*	*p*	Collinearity diagnosis	
	*B*	Standard Error	*Beta*			VIF	Tolerance	*B*	Standard Error	*Beta*			VIF	Tolerance
Constant	55.860	4.642	–	12.033	0.000**	–	–	30.373	3.598	–	8.442	0.000**	–	–
Age	1.081	0.171	0.265	6.304	0.000**	6.645	0.150	0.762	0.133	0.304	5.729	0.000**	6.645	0.150
Gender	−2.543	0.472	−0.097	−5.387	0.000**	1.230	0.813	0.918	0.366	0.057	2.508	0.012*	1.230	0.813
BMI	1.286	0.234	0.190	5.499	0.000**	4.500	0.222	0.468	0.181	0.113	2.583	0.010**	4.500	0.222
RHR	0.066	0.022	0.052	2.990	0.003**	1.151	0.869	0.182	0.017	0.233	10.556	0.000**	1.151	0.869
LC	0.003	0.000	0.296	9.350	0.000**	3.770	0.265	0.001	0.000	0.108	2.704	0.007**	3.770	0.265
*R* ^2^	0.510							0.219						
Adjusted *R*^2^	0.509							0.217						
*F*	*F* (5,1842) = 383.782, *p* = 0.000							*F* (5,1842) = 103.438, *p* = 0.000						
D-W	1.949							1.919						

Dependent variable: SBP, DBP. *p < 0.05, **p < 0.01, CC, Chest circumference; RHR, Resting Heart Rate; LC, Lung capacity.

**Table 8:** Linear regression analysis of obesity blood pressure.

Obesity SBP linear regression analysis results (*n* = 1,363)								Obesity DBP linear regression analysis results (*n* = 1,363)						
	Non-standardized coefficient		Standardized coefficient	*t*	*p*	Collinearity diagnosis		Non-standardized coefficient		Standardized coefficient	*t*	*p*	Collinearity diagnosis	
	*B*	Standard Error	*Beta*			VIF	Tolerance	*B*	Standard Error	*Beta*			VIF	Tolerance
Constant	65.401	3.539	–	18.481	0.000**	–	–	24.922	2.882	–	8.649	0.000**	–	–
Age	1.206	0.157	0.304	7.675	0.000**	3.791	0.264	0.725	0.128	0.267	5.664	0.000**	3.791	0.264
Gender	−2.130	0.577	−0.083	−3.693	0.000**	1.221	0.819	1.042	0.470	0.059	2.220	0.027*	1.221	0.819
BMI	0.609	0.107	0.141	5.708	0.000**	1.486	0.673	0.593	0.087	0.202	6.828	0.000**	1.486	0.673
RHR	0.111	0.027	0.089	4.128	0.000**	1.120	0.893	0.219	0.022	0.258	10.062	0.000**	1.120	0.893
LC	0.003	0.000	0.302	7.714	0.000**	3.708	0.270	0.000	0.000	0.038	0.808	0.419	3.708	0.270
*R* ^2^	0.439							0.203						
Adjusted *R*^2^	0.437							0.200						
*F*	*F* (5,1357) = 212.273, *p* = 0.000							*F* (5,1357) = 69.044, *p* = 0.000						
D-W	1.875							1.948						

Dependent variable: SBP, DBP. *p < 0.05, **p < 0.01, CC, Chest circumference; RHR, Resting Heart Rate; LC, Lung capacity.

The derived blood pressure prediction models for different BMI categories in this study are as follows:

For normal-weight children:

SBP = 53.064 + 1.429*Age-3.101*Gender +1.106*BMI + 0.108*RHR + 0.004*LC

DBP = 31.473 + 1.092*Age + 0.678*Gender +0.257*BMI + 0.175*RHR + 0.001*LC

For underweight children:

SBP = 85.761 + 2.457*age – 3.379*Gender – 1.195*BMI + 0.054*RHR + 0.002*LC

DBP = 38.947 + 1.744*age – 0.267*Gender – 0.621*BMI + 0.173*RHR + 0.001*LC

For obese children:

SBP = 65.401 + 1.206*Age-2.130*Gender +0.609*BMI + 0.111*RHR + 0.003*LC

DBP = 24.922 + 0.725*Age + 1.042*Gender +0.593*BMI + 0.219*RHR + 0.000*LC

For overweight children:

SBP = 55.860 + 1.081*Age-2.543*Gender +1.286*BMI + 0.066*RHR + 0.003*LC

DBP = 30.373 + 0.762*Age + 0.918*Gender +0.468*BMI + 0.182*RHR + 0.001*LC

Note: RHR: Resting Heart Rate; LC: Lung capacity; SBP: Systolic blood pressure; DBP: Diastolic blood pressure.

#### 3.4.2 K-fold cross-validation

In this study, the K-fold cross-validation method was used with *K* = 5 to analyze the SBP and DBP prediction models of children with different weight levels. The results (Table 9) indicate that the SBP models for normal weight and overweight children demonstrate strong explanatory power, explaining 49.4 and 51.0% of the variability, respectively. In contrast, the explanatory power of the DBP models is generally lower across all weight categories, with the highest explanatory power at 44.2% observed in obese children.

**Table 9:** K-fold cross-validation.

Metric	Detail	SBP model	DBP model
K-fold cross-validation configuration	Predictors	5	
	Pre-processing	None	
	Resampling method	5-fold cross-validation	
	Predictors	5	
	Pre-processing	None	
	Resampling method	5-fold cross-validation	
	Intercept	Held constant at TRUE	
Under weight	Number of samples	164	164
	Sample sizes	131, 131, 131, 131, 132	132, 131, 130, 132, 131
	RMSE	10.59613	8.755103
	R-squared	0.2871954	0.2076471
	MAE	8.915467	6.944548
Normal	Number of samples	7882	7882
	Sample sizes	6307, 6305, 6304, 6306, 6306	6306, 6306, 6305, 6305, 6306
	RMSE	9.628235	7.440449
	R-squared	0.4942258	0.2543182
	MAE	7.680205	5.842462
Obese	Number of samples	1363	1363
	Sample sizes	1090, 1091, 1090, 1091, 1090	1092, 1090, 1090, 1090, 1090
	RMSE	9.414682	9.392665
	R-squared	0.4352527	0.442462
	MAE	7.211468	7.21842
Over weight	Number of samples	1848	1848
	Sample sizes	1479, 1478, 1478, 1479, 1478	1479, 1477, 1478, 1479, 1479
	RMSE	9.14805	7.087499
	R-squared	0.5101171	0.2170035
	MAE	7.148291	5.564969

Regarding model accuracy, the DBP model for normal weight children has a lower root mean square error (RMSE), while the models for overweight and obese children have smaller errors in predicting SBP. This result reflects the different dynamics of blood pressure among different weight categories. The blood pressure prediction models for different weight categories also show significant differences in accuracy and explanatory power, implying that future predictions of blood pressure in specific weight categories of adolescent children may require more refined predictive methods and optimization of model parameters tailored to specific weight groups.

#### 3.4.3 Independent sample validation

During the independent sample validation process, 120 test samples were randomly split and inserted into the prediction model formula. The predicted blood pressure was then cross-validated with the actual blood pressure. The prediction accuracy of SBP and DBP for adolescent children of different weight categories (normal, obese, overweight) was evaluated using three metrics: mean squared error (MSE), root mean square error (RMSE), and mean absolute error (MAE). The findings (Table 10) show that the obese population has the lowest error metrics among all groups, indicating the model’s precision in predicting blood pressure for this group. The prediction errors for the normal weight population are relatively higher, especially in DBP prediction. The prediction errors for the overweight population fall between those of the normal and obese populations. The blood pressure prediction model performs optimally for the obese population, while the accuracy of the model for the normal weight population is relatively lower. These differences may be related to variations in physiological characteristics under different weight statuses, suggesting that future model development should consider specific influencing factors related to weight categories.

**Table 10:** Independent sample cross-validation.

Indicator types	Normal		Obese		Overweight	
	SBP	DBP	SBP	DBP	SBP	DBP
MSE	78.04751	50.59709	42.54946	28.98169	69.51828	45.8415
RMSE	8.83445	7.113163	6.522994	5.383464	8.337762	6.770635
MAE	6.933713	5.548267	5.842115	4.566377	7.085086	5.603714

## 4 Discussion

Currently, childhood hypertension has become an important challenge in the field of public health (34, 35). Preventing and controlling hypertension has been widely recognized as one of the key topics in contemporary medical research. Since the 1970s, countries around the world have been committed to studying blood pressure-related issues in adolescents and children, continuously striving to promote youth blood pressure health (36).

In this study, we designed and validated a model for predicting SBP and DBP in children based on different weight categories. The model’s construction considered factors such as Age, Gender, BMI, RHR, and LC, and the model’s significance was verified through F-tests. The process of model construction and validation showed that Age, Gender, BMI, and other factors have statistically significant effects on children’s blood pressure, with all models having D-W values close to 2, further indicating the absence of autocorrelation in the model. Results from K-fold cross-validation revealed that the SBP model exhibits strong explanatory power for normal weight and overweight children. Although the explanatory power of DBP models across different weight categories is generally lower, the highest explanatory power was observed in obese children. Model validation using independent samples demonstrated that the blood pressure prediction model performs best for obese children. These research findings not only validate the effectiveness of our established blood pressure prediction model but also highlight the importance of considering children’s weight categories in future developments and adjustments to blood pressure management strategies.

Amid the COVID-19 pandemic and other large-scale epidemiological contexts, the demand for health monitoring in schools has significantly increased. At this time, a simple and fast blood pressure prediction model becomes particularly important. The data required for this model, including Age, Gender, BMI, RHR, and LC, can be easily obtained through routine health checks without the need for blood pressure measurement devices, greatly reducing operational complexity and economic burden. In the context of a large-scale epidemic, where minimizing face-to-face interactions is necessary, teachers or school health workers can conveniently conduct health screenings without the need for close contact with students, ensuring rapid health monitoring in large student populations, significantly improving monitoring efficiency, and allowing more students to benefit from regular blood pressure assessments in a timely manner. This not only supports health management during the pandemic but also strengthens schools’ rapid response capabilities to public health events.

This study further emphasizes the year-on-year increase in blood pressure among children and adolescents during their growth and development process. This result is consistent with findings from past research. Wang (37) observed in a three-year longitudinal study that average blood pressure in both boys and girls tends to increase with Age (38). Cheng (33), through an 8-year observation period with 71,468 participants, found that SBP typically continues to rise with Age, while DBP tends to decrease after reaching middle age. This pattern of blood pressure changes with Age has been validated in multiple populations and is considered a common phenomenon in the aging process (39). Li (40), through a cross-sectional survey of health conditions in China, evaluated the association between body mass index, lean body mass percentage, visceral fat level, and blood pressure in each Age group. The study highlights the dynamic changes in SBP and DBP throughout the lifespan, which are closely related to physiological changes in arterial walls and the cardiovascular system brought about by Age-related alterations (41). The increase in blood pressure is a concomitant phenomenon of children’s growth and development. For children, the influence of age on blood pressure is an inevitable result, indicating certain regularities in blood pressure changes among children.

Age, as a continuously increasing factor, tends to lead to a rise in blood pressure with age (25). The growth of the heart and the increase in cardiac output are key factors in the impact of age on blood pressure (42). As children age, their hearts gradually mature, their cardiac cavity volume increases, and their cardiac output correspondingly increases. This physiological change results in more blood being pumped into the arterial system during systole, leading to an increase in systolic blood pressure (SBP). At the same time, the stroke volume of the heart also increases. For children, this is a normal physiological development process. The autonomic nervous system of adolescents gradually stabilizes in controlling heart rate, vascular tension, and blood pressure (43). Increasing age is accompanied by a more coordinated and mature sympathetic and parasympathetic nervous system, which helps maintain the stability of the cardiovascular system (44). Additionally, increases in weight and height directly affect the workload of the heart and the demands of the vascular system (45). Weight gain may lead to increased cardiac load, while height increase requires more blood to maintain normal circulation.

The study observed that within the same age group of children, blood pressure levels exhibit significant differences due to Gender variations. This phenomenon may be related to specific physiological and hormonal differences associated with Gender, affecting blood pressure regulation mechanisms. Gianvincenzo (46) conducted a cross-sectional survey of 4,514 pre-adolescent children and found that Gender differences influence blood pressure in adolescent children, particularly in the 6–11 pre-adolescent age group, where this phenomenon is especially significant (47). Typically, boys have higher blood pressure than girls, and as Age increases, this difference becomes more pronounced. Additionally, blood pressure variations are also influenced by factors such as obesity, sleep habits, unhealthy behaviors, and social factors (48).

Gender differences may stem from differences in physiological structure and hormone levels (49). Boys experience an increase in testosterone levels during puberty, while girls undergo different changes in hormone levels (50). The increase in testosterone levels may lead to increased muscle mass, increased blood volume, and increased cardiac output in boys, thereby affecting blood pressure (51). Estrogen in females may affect blood pressure levels through pathways such as regulating vascular wall elasticity and endothelial function, as well as regulating the renin-angiotensin system (52). During the growth and development process of children, their hormone levels and blood pressure may change, which is related to gender (53). Furthermore, during development, girls in the same age group have higher subcutaneous fat areas than boys, while visceral fat in females is lower than in males, resulting in a lower risk of hypertension for females under the same BMI conditions. The development and physiological characteristics of the cardiovascular system are also associated with gender differences. Differences exist between boys and girls in heart size, vascular elasticity, cardiac output, and other physiological characteristics, which may play important roles in the formation of blood pressure levels (54). Due to gender differences, lifestyle and behavioral habits differ between boys and girls of the same age group, including differences in diet, exercise, and weight management, which may affect blood pressure levels (55).

This study confirms a positive correlation between children’s body mass index (BMI) and their blood pressure levels. As BMI increases, both DBP and SBP in children show an upward trend, indicating that an increase in weight leads to higher blood pressure, thereby increasing the risk of cardiovascular diseases. This is consistent with numerous previous research findings, suggesting that obesity is a significant risk factor for blood pressure changes (25). Previous studies have indicated that adiponectin, a fat tissue-specific hormone, is significantly lower in the plasma of hypertensive individuals compared to healthy individuals with normal blood pressure levels (56, 57). It has been confirmed that plasma adiponectin levels are much lower in overweight and obese individuals compared to normal individuals, leading to increased blood pressure in obese patients (58). Recent research has demonstrated that obesity leads to overactivation of the sympathetic nervous system, resulting in elevated blood pressure (25, 59). Typically, obesity is accompanied by excessive visceral fat accumulation, which not only damages the function of pancreatic beta cells, leading to inflammation, oxidative stress, and decreased glucose metabolism, but also causes abnormal blood lipids and increased blood pressure (60).

As a measure of body mass, BMI is positively associated with increased risk of high blood pressure in adolescents (61). This association reveals a close physiological connection between adolescent obesity and hypertension (62, 63). Under conditions of obesity, there is an accumulation of fat in the body, leading to a significant increase in cardiovascular burden. Obesity not only requires the heart to exert greater force to pump blood, but also may disrupt other parts of the cardiovascular system (64). This excessive burden may accelerate the development of arteriosclerosis, increase vascular resistance, and thereby lead to elevated blood pressure (65). Additionally, factors associated with high BMI and hypertension are related to chronic inflammation associated with obesity (66). Previous research has shown that under conditions of obesity, the inflammatory response in adipose tissue increases, which may trigger systemic chronic inflammation (67). Chronic inflammation may damage blood vessel walls, making them more prone to constriction and loss of elasticity, thus adversely affecting blood flow and leading to hypertension. Moreover, obesity also increases the risk of other cardiovascular diseases such as coronary heart disease, stroke, and heart disease (45), all of which are closely related to blood pressure abnormalities. Therefore, high BMI as a potential cardiovascular risk factor may exacerbate the occurrence and development of these diseases.

The blood pressure of adolescents is closely related to RHR and lung function, and this finding has important clinical significance for monitoring children’s health. Numerous past studies have shown that changes in RHR can significantly predict blood pressure levels, while good lung function is associated with lower blood pressure levels. Past research has indicated that RHR has a significant impact on pulmonary artery blood flow dynamics (68). Zhang (69) used a support vector machine regression algorithm to develop an efficient blood pressure prediction method, utilizing machine learning techniques for joint training and predicting the relationship between physiological indicators (RHR, blood oxygen saturation, etc.) and blood pressure (70). Acceleration in RHR often accompanies an increase in blood pressure, and the strength of lung function is closely related to blood pressure regulation.

The increase in RHR typically reflects an increased cardiac workload, which may be closely related to hypertension. RHR is a direct indicator of cardiac pumping efficiency; an increase in RHR usually means an increase in the amount of blood pumped by the heart per minute, directly affecting arterial blood pressure levels (71). Acceleration in RHR temporarily increases ventricular filling and cardiac output, thereby raising DBP. An increase in RHR also affects the length of the cardiac diastole, which may lead to changes in SBP (72). Furthermore, changes in RHR are often considered a response to cardiovascular activity pressure, reflecting the level of activity of the sympathetic nervous system. When an individual is under stress or at risk of cardiovascular disease, activation of the sympathetic nervous system can lead to an increase in RHR, thus affecting blood pressure (73). Epidemiological studies suggest that long-term trends in RHR can serve as an important indicator for predicting hypertension and other cardiovascular diseases. The association between higher RHR and the development of hypertension has been confirmed in several large-scale cohort studies (74).

Good lung function supports effective oxygen exchange and blood circulation in the body, aiding in reducing cardiovascular pressure and controlling blood pressure. Lung function directly impacts the intake of oxygen and the elimination of carbon dioxide, which are crucial for maintaining blood oxygen saturation and acid–base balance (75). Lung dysfunction is common in chronic obstructive pulmonary disease or asthma, leading to hypoxemia and hypercapnia. These conditions can affect the cardiovascular system through neural and hormonal response mechanisms, causing blood vessel constriction and increasing blood pressure (76). Hypoxemia activates the sympathetic nervous system and adrenaline release, resulting in accelerated RHR and vascular constriction, which can lead to elevated blood pressure. Additionally, obesity is a common risk factor for decreased lung function and hypertension. Obesity not only reduces lung capacity and limits lung expansion but also affects blood pressure regulation through various mechanisms, including changes in hormone activity (such as insulin resistance and abnormal adipokine secretion). Inflammation plays a bridging role in these conditions, contributing to an increase in systemic inflammation levels and subsequent hypertension. Some large-scale cohort studies have found that even after excluding smoking as a major risk factor, decreased lung function remains closely associated with an increased risk of hypertension (76, 77).

By understanding the effects of factors such as age, gender, RHR, and BMI on blood pressure, we can better formulate personalized health plans and intervention measures to help prevent hypertension and its related complications, thereby improving overall health levels. Additionally, for healthcare practitioners, these findings provide important evidence for developing more effective treatment regimens and health policies to promote public health and well-being.

### 4.1 Limitations of the study

1. The sample used in this study is from children aged 7–17 population in City N, China. Children from different regions, races, and cultural backgrounds may have different lifestyles and genetic backgrounds, thus possibly different impacts on blood pressure.
2. This study only considered the effects of factors such as age, gender, and BMI on blood pressure, while other factors such as genetic factors, environmental factors, dietary habits, and lifestyle may also have important influences on blood pressure formation and changes, which deserve further research and exploration.
3. This study used a cross-sectional design, which cannot capture changes and developmental trends over time. Future longitudinal study designs may reveal the long-term effects of age, gender, and BMI on blood pressure changes.
4. Due to the restrictions and closed management measures during the COVID-19 period, the representativeness of the sample may be affected. Some populations may not be able to participate in the survey due to isolation or restrictions, which may result in an insufficiently comprehensive sample.

## 5 Conclusion

This study confirms a significant association between BMI and blood pressure among adolescents aged 7 to 17 during the COVID-19 period. The study involved 11,433 primary and secondary school students, with 11,287 valid samples after data screening. The analysis results indicate that Age, Gender, and BMI have a significant impact on both SBP and DBP. By constructing multiple regression analyses, the study elucidates how factors such as Age, Gender, BMI, RHR, and LC collectively influence blood pressure. The study establishes blood pressure prediction models for adolescents of different obesity levels and utilizes K-fold cross-validation and independent sample cross-validation to assess the explanatory power of the included indicators on blood pressure changes, predicting blood pressure in children of different weight levels, thus providing a scientific basis for targeted health plans and interventions.

These findings are crucial for screening adolescents for hypertension and managing cardiovascular health, especially during large-scale infectious disease outbreaks. Effective prediction models can assist healthcare professionals in better monitoring and managing adolescent blood pressure issues during pandemics, enabling the formulation of more effective treatment plans and health policies to address health challenges and promote the health and well-being of adolescents.

## Data availability statement

The original contributions presented in the study are included in the article/supplementary material, further inquiries can be directed to the corresponding authors.

## Ethics statement

The studies involving humans were approved by the Ethics Committee of the Sports Human Body Science Research Institute of Nanjing Sport Institute. The studies were conducted in accordance with the local legislation and institutional requirements. The participants’ legal guardian/next of kin provided their written informed consent to participate in this study.

## Author contributions

SM: Conceptualization, Funding acquisition, Investigation, Formal analysis, Methodology, Software, Visualization, Writing – original draft. GQ: Investigation, Methodology, Writing – original draft, Data curation. KX: Investigation, Validation, Writing – review & editing. HX: Validation, Methodology, Writing – original draft. HZ: Data curation, Formal analysis, Writing – review & editing. XG: Conceptualization, Funding acquisition, Investigation, Writing – review & editing.
